# Red and near-infrared photobiomodulation for burn, hypertrophic, and post-surgical scars: a scoping review of clinical trials

**DOI:** 10.1007/s10103-026-04875-8

**Published:** 2026-04-24

**Authors:** Simonetta I. Gaumond, Emilee M. Dreifus, Amisha Mittal, Ariel E. Eber

**Affiliations:** 1 Dr. Phillip Frost Department of Dermatology and Cutaneous Surgery, University of Miami, Miami, USA; 2 Department of Biochemistry and Molecular Biology, University of Miami, Miami, USA

**Keywords:** Photobiomodulation, Red light therapy, Near-infrared therapy, Light-emitting diode (LED), Low-level laser therapy (LLLT), Scar, Scarring, Hypertrophic scars, Burn scars, Post-surgical scars

## Abstract

Purpose: Scars may lead to pain, pruritus, functional impairment, and cosmetic concern despite standard therapies. Photobiomodulation (PBM) delivered by light-emitting-diode (LED) or low-level laser therapy (LLLT) has emerged as a non-invasive option to modulate scar remodeling. However, available data derives from small, heterogenous studies. This scoping review evaluates whether red and near-infrared PBM improves clinical outcomes in burn, hypertrophic, and post-surgical scars and summarizes treatment parameters used. Methods: PubMed, Embase, and MEDLINE were searched through October 2025 for randomized or prospective clinical studies in which red (633-670nm) or near-infrared (808-830nm) LED or LLLT was used as monotherapy or where its contribution could be independently assessed. Seven studies (n=297) met inclusion criteria. Data on scar type, wavelength, fluence, regimen, outcomes, and adverse events were extracted. Results: Red LED improved VSS scores, pigmentation, and thickness in pediatric and adult burn scars, with greater benefits observed in scars < 12 months. In post-surgical scars, red LED reduced induration and improved POSAS scores, with a biphasic dose-response favoring moderate fluence levels. Near-infrared PBM improved color and elasticity in hypertrophic scars and reduced thickness, malleability, pain, and pruritus following hernia repair, thyroidectomy, and blepharoplasty. Across studies, PBM was well tolerated; transient erythema and warmth were most common, with rare blistering at higher fluences. Conclusion: Red and near-infrared PBM appears to be a safe, non-invasive adjunct to standard scar management, particularly for early burn and post-operative scars. Larger controlled trials are needed to define optimal wavelength, fluence, timing, and treatment parameters across diverse scar phenotypes and skin phototypes. Clinical Trial Registration: Not applicable.

## Introduction

Scars, whether from burns, surgery, or trauma, can lead to pain, pruritus, restricted mobility, and substantial psychosocial burden [1]. Despite the availability of silicone therapy, pressure garments, intralesional corticosteroids, and laser-based interventions, outcomes remain variable, and many patients continue to experience persistent symptoms or cosmetically distressing changes [2]. As a result, there is increasing interest in non-invasive modalities that modulate the wound-healing cascade without introducing thermal injury or procedural downtime.

Photobiomodulation (PBM) using red (620–700 nm) and near-infrared (700-1,440 nm) wavelengths has emerged as a promising adjunct for scar management [3]. Delivered through light-emitting diodes (LEDs) or low-level laser therapy (LLLT), PBM activates cytochrome c oxidase and enhances mitochondrial function, leading to downstream modulation of inflammatory and profibrotic pathways implicated in scar remodeling [4–7].

**Fig. 1:**
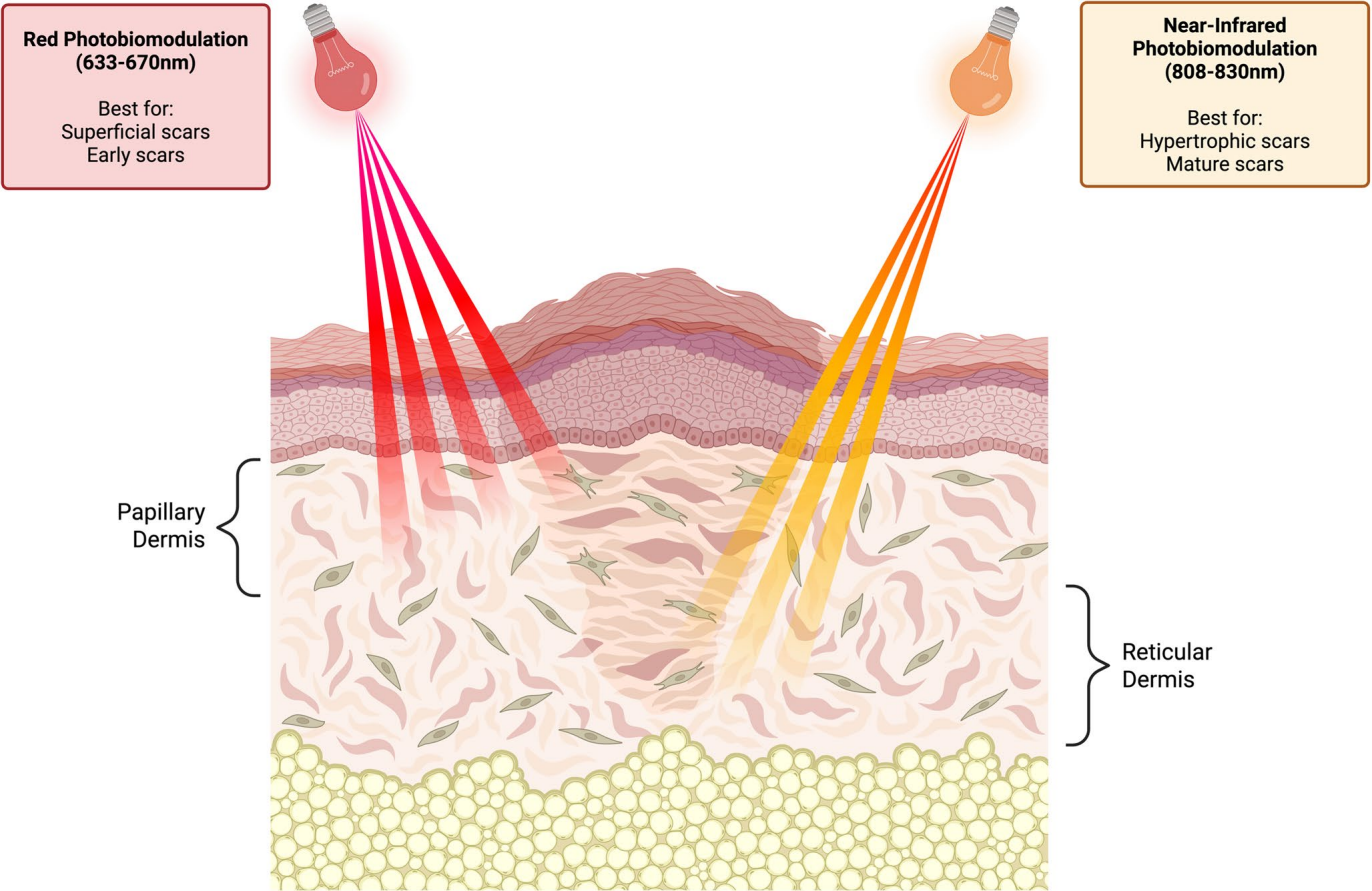
Wavelength-specific penetration depth of red (633–670 nm) and near-infrared (808–833 nm) photobiomodulation. Red PBM primarily affects superficial dermal layers and is best suited for early, superficial, and diffuse scars. Near-infrared PBM penetrates more deeply into the dermis and is better suited for hypertrophic or mature scars. The differential depth of penetration provides a mechanistic rationale for wavelength-tailored scar treatment

Although both LEDs and LLLT deliver red and near-infrared wavelengths for PBM, their physical properties differ in ways that may influence clinical outcomes. LEDs emit non-coherent, broad-beam light with a wide treatment coverage and minimal heat generation [8], making them suitable for larger or irregular surfaces such as burn scars. In contrast, LLLT devices emit coherent, collimated light with greater photon density and deeper tissue penetration [8], allowing more targeted delivery for thicker or focal hypertrophic scars (Fig. 1).

Over the past two decades, clinical evidence supporting PBM for scars has expanded across burn, hypertrophic, and post-surgical settings, with emerging dose-response and wavelength-specific patterns. However, heterogeneity in study design, treatment parameters, timing of intervention, and outcome reporting has limited standardization and slowed clinical adoption. To address these gaps, this scoping review synthesizes clinical trials evaluating red and near-infrared PBM, via LED or LLLT, for burn, hypertrophic, and post-surgical scars, and aims to identify treatment parameters associated with optimal clinical improvement and safety. By integrating evidence across diverse scar etiologies/phenotypes, this review provides a foundational framework for the standardized use of PBM as a non-invasive adjunct in scar management.

## Methods

A scoping review was performed to assess the safety, treatment parameters, and clinical efficacy of red and near-infrared PBM delivered through LED or LLLT for cutaneous scars. A comprehensive search of PubMed, Embase, and MEDLINE was conducted through October 2025 using combinations of the terms “photobiomodulation,” “red,” “near-infrared,” “light-emitting diode (LED),” “low-level laser therapy (LLLT),” and “scar,” combined with Boolean operators.

Inclusion criteria comprised randomized or prospective clinical studies in human participants with scars of any etiology treated with red or near-infrared PBM using LED or LLLT, with at least one reported clinical or patient-reported scar outcome. Exclusion criteria included in vitro or animal studies, uncontrolled studies, conference abstracts without extractable outcome data, non-English publications, and studies in which PBM was combined with other modalities without the ability to independently assess its contribution.

Extracted variables included study design, sample characteristics, scar type, wavelength and fluence parameters, treatment regimen, outcome measures, follow-up duration, and reported adverse events. When available, cumulative photobiomodulation exposure was calculated as the product of fluence per session and the total number of treatment sessions.

The initial database search yielded 118 records. After removal of duplicates and screening of titles and abstracts, 24 studies underwent full-text review. Seven studies, encompassing 297 patients, met the inclusion criteria and were included in the final synthesis.

## Results

### Red LED (633–670 nm)

#### Burn scars

Red LED therapy has demonstrated meaningful improvements in post-burn scar remodeling and symptomatic relief across pediatric and adult populations (Table 1). In a randomized split-scar trial, Alsharnoubi et al. treated 15 children (aged 2–10 years) with 633 nm LED (16 J/cm^2^ per session) administered twice weekly for 12 weeks (24 sessions; cumulative dose 384 J/cm^2^) [9]. Although both treated and untreated halves improved over time (*p* = 0.001), LED-treated areas showed significantly greater reductions in Vancouver Scar Scale (VSS) scores (*p* = 0.003), pigmentation (*p* = 0.002), and height (*p* = 0.003) relative to controls.

**Table 1:** Clinical Studies Using Red PBM for the Treatment of Scars. Summary of clinical trials using red LED (633–670 nm) post-burn and post-surgical scars

Scar type	Study	Study design	Participant characteristics	Fluence per session	Number of sessions	Cumulative dose	Outcomes	Adverse events
Burn scars	Alsharnoubi et al.	RCT, split-scar, placebo-controlled	15 children (2–10 y/o)	16 J/cm²	24	384 J/cm²	↓ VSS score in treated vs. control (*p* = 0.003); ↓ pigmentation (*p* = 0.002), ↓ scar height (*p* = 0.003)	None
	Gaida et al.	RCT, split-scar, placebo-controlled	19 patients (18–77 y/o)	4 J/cm²	16	64 J/cm²	↓ VSS from 7.10 to 1.42 (LED) vs. 5.86 to 5.40 (placebo)	None
Surgical Scars	Kurtti et al.	RCT, split-face, placebo-controlled, dose-ranging	30 women (≥ 18 y/o)	20 J/cm² (low), 36 J/cm² (moderate), 53 J/cm² (high)	9	180 J/cm² (low), 320 J/cm² (moderate), 480 J/cm² (high)	At 6 months: moderate fluence ↓ induration by 77.8% vs. 50% (control); low fluence ↓ by 62.6% vs. 40% (control); no group-wise significance	Localized bulla formation (6.7%), localized facial swelling (3.3%), erythema (100%), and warmth (100%)

Similarly, Gaida et al. evaluated 670 nm LED (4 J/cm^2^ per session) administered twice weekly for eight weeks (16 sessions; cumulative dose 64 J/cm^2^) in 19 adults with post-burn scarring using a split-scar design [10]. LED-treated areas demonstrated substantial VSS improvement (7.10 ± 3.07 to 1.42 ± 1.67), whereas placebo-treated sites showed minimal changes (5.86 ± 2.71 to 5.40 ± 2.66). Symptoms also improved significantly, with pain decreasing from 3.81 ± 3.07 to 1.42 ± 1.67 and pruritus from 4.86 ± 3.26 to 1.31 ± 1.88. Scars younger than 12 months responded more robustly, suggesting a therapeutic window during early scar remodeling.

#### Post-surgical scars

Evidence for red LED therapy in post-surgical scar modulation is most developed in cosmetic surgery (Table 1). Kurtti et al. conducted a randomized split-face trial in 30 facelift patients, initiating 633 nm LED treatment one week after surgery [11]. Patients were assigned to low (180 J/cm^2^), moderate (320 J/cm^2^), or high (480 J/cm^2^) cumulative dose fluence regimens, administered three times weekly for three weeks, with the contralateral side serving as control. At six months, the moderate-fluence group achieved the greatest improvement, with a 77.8% reduction in induration on the treated side compared to 50% on the placebo side. The low-fluence group also demonstrated benefit, achieving a 62.5% reduction compared to 40% in placebo-treated areas, while the high-fluence regimen produced no benefit over control (50.0% vs. 71.4%), reflecting a biphasic dose-response pattern. These findings were supported by Patient and Observer Scar Assessment Scale (POSAS) scores, in which the moderate-fluence group again demonstrated the largest improvement (57.9% vs. 45.5% for low-fluence, and 24.2% for placebo). Adverse effects were notable, with three patients (10%) experiencing localized bulla formation or swelling at the treatment site. Transient erythema and warmth were universal but resolved within 24 h.

### Near-infrared LED (808–833 nm)

#### Hypertrophic scars

Clinical evidence evaluating near-infrared LED therapy for hypertrophic scars remains limited but promising (Table 2). Freitas et al. conducted a randomized, placebo-controlled split-scar trial in 17 adults with hypertrophic scars on the trunk and extremities using an 808 nm LED device (4 J/cm^2^ per session) [12].Treatments were administered three times per week for five weeks (15 sessions; cumulative dose 60 J/cm^2^), with one scar randomized to active LED and another to placebo within each patient. At follow-up, LED-treated scars demonstrated significant improvements in color (*p* = 0.004) and elasticity (*p* = 0.016), whereas placebo-treated scars showed minimal change. Clinical reductions of 22.2% in scar length and 44% in scar width was observed in LED-treated areas. Notably, the greatest improvements occurred in older scars exceeding 24 months in age, suggesting that near-infrared LED may offer therapeutic benefit even beyond the early proliferative phase of scar maturation.

**Table 2:** Clinical Studies Using Near-Infrared PBM for the Treatment of Scars. Summary of clinical trials using near-infrared LED or LLLT (808–830 m) for hypertrophic and post-surgical scars

Scar Type	Study	Study design	Participant characteristics	Fluence per session	Number of sessions	Cumulative dose	Outcomes	Adverse events
Hypertrophic scars	Freitas et al.	RCT, split-scar, placebo-controlled	17 patients (18–25 y/o)	4 J/cm²	15	60 J/cm²	↓ Scar color (*p* = 0.004), ↑ elasticity (*p* = 0.016); scar length ↓ 22.2%, width ↓ 44%	None
Surgical scars	Carvaldo et al.	RCT, parallel, placebo-controlled	28 inguinal hernia repair patients (37–57 y/o)	13 J/cm²	4	52 J/cm²	↓ VSS (2.14 vs. 4.85; *p* = 0.0002), ↓ scar thickness (0.11 cm vs. 0.19 cm), ↑ malleability (0.14 vs. 1.07; both *p* < 0.05)	None
	Kim et al.	RCT, double-blind, sham-controlled	43 thyroidectomy patients (≥ 18 y/o)	4.5 J/cm²	28	126 J/cm²	↑ satisfaction (*p* = 0.008), ↑ GAS (*p* = 0.002), ↓ pain (*p* = 0.004), ↓ VSS (*p* = 0.004)	None
	Ye & Xiang	RCT, parallel, standard care vs. LED	145 blepharoplasty patients (≥ 18 y/o)	60 J/cm²	4	240 J/cm²	↑ wound healing (96.6% vs. 86.8%, *p* < 0.05), ↓ swelling, pain, anxiety (*p* < 0.05)	None

#### Post-surgical scars 

Near-infrared LED photobiomodulation has shown consistent benefit across multiple post-operative settings, including general surgery, endocrine surgery, and oculoplastic procedures (Table 2). Carvaldo et al. evaluated 830 nm LED therapy (13 J/cm^2^ per session) in 28 patients following inguinal hernia repair, administered on postoperative days 1, 3, 5, and 7 (4 sessions; cumulative dose 52 J/cm^2^) [13]. By the six-month follow-up, LED-treated scars demonstrated significantly lower VSS scores (2.14 ± 1.51 vs. 4.85 ± 1.87; *p* = 0.0002), reduced thickness (0.11 cm vs. 0.19 cm; *p* < 0.05), and improved malleability (0.14 vs. 1.07; *p* < 0.05) compared with controls.

A randomized, double-blind, placebo-controlled trial by Kim et al. examined daily home-use 830 nm LED therapy (4.5 J/cm^2^ per session) initiated one week after surgery and continued for four weeks (approximately 28 sessions; cumulative dose 126 J/cm^2^), in 43 thyroidectomy patients [14]. At six months, the LED group reported significantly higher satisfaction (*p* = 0.008) and global assessment scores (*p* = 0.002), alongside lower pain (*p* = 0.005) and VSS scores (*p* = 0.004) compared with the placebo group. Three-dimensional imaging favored the LED treatment in color, height, pigmentation, and vascularity, although these differences did not reach statistical significance. The treatment was well tolerated, and no adverse events were reported.

Further supporting these findings, Ye & Xiang conducted a randomized trial in 145 blepharoplasty patients comparing standard postoperative care with adjunctive 830 nm LED therapy (60 J/cm^2^ per session) on postoperative days 1, 2, 3, 7 (4 sessions; cumulative dose 240 J/cm^2^) [15]. By day seven, the LED-treated group showed notably higher incision-healing rates (96.6% vs. 86.8%; *p* < 0.05), along with significant reductions in swelling, pain, and anxiety (all *p* < 0.05). No adverse events were reported.

## Discussion

Across seven randomized or prospective studies, both red and near-infrared wavelengths demonstrated improvements in scar pigmentation, pliability, thickness, elasticity, and patient-reported symptoms, and were consistently well tolerated. Despite heterogeneity in devices and treatment regimens, several clinically meaningful patterns emerged that may guide wavelength selection and treatment timing.

Red LED therapy showed the most pronounced benefits in early burn scars and recent post-surgical scars. In pediatric and adult burn populations, scars younger than 12 months responded more robustly, suggesting that red wavelengths may be particularly effective during the early remodeling phase when fibroblast activity and collagen turnover are most active [16]. In cosmetic postoperative scars, a biphasic dose-response was observed, with moderate fluence producing superior improvements in induration and POSAS scores compared with both lower and higher doses. This aligns with established PBM principles in which insufficient fluence yields subtherapeutic stimulation, whereas excessive fluence may inhibit mitochondrial responsiveness [17]. Together, these findings suggest that red LED is best suited for early, actively remodeling scars and should be delivered within a moderate therapeutic window to maximize clinical benefit (Table 3).

**Table 3:** Clinical considerations for red and near-infrared photobiomodulation in scar management. PBM, photobiomodulation; VSS, Vancouver Scar Scale; POSAS, Patient and Observer Scar Assessment Scale

Wavelength	Typical indication	Scar stage	Reported clinical benefits	Practical considerations
Red PBM (633–670 nm)	Burn scars, early post-surgical scars	Early remodeling phase (< 12 months)	Improved pigmentation, reduced scar height, improved VSS and POSAS scores	Moderate fluence appears most effective; excessive fluence may reduce therapeutic response
Near-Infrared PBM (808–830 nm)	Hypertrophic scars, mature surgical scars	Mature scars (> 12–24 months)	Improved elasticity, malleability, scar color, pain and pruritus reduction	Greater dermal penetration allows treatment of thicker or deeper scars

Near-infrared LED therapy generated consistent improvements across hypertrophic and post-surgical scars, including scars older than two years old and scars located in deeper anatomic sites. Improvements in pigmentation, elasticity, thickness, malleability, and patient satisfaction were observed across inguinal hernia repair, thyroidectomy, and blepharoplasty scars. The efficacy of near-infrared LED in older hypertrophic scars may reflect the deeper tissue penetration of 808–830 nm wavelengths, allowing modulation of fibroblast behavior and collagen remodeling even after the proliferative phase has subsided [18]. The success of a self-administered 830 nm device in thyroidectomy patients further highlights the practicality and accessibility of PBM as an adjunctive treatment in routine postoperative care, although home use of medical devices using intense light should be considered with care.

Mechanistically, both red and near-infrared wavelengths activate cytochrome c oxidase, increase mitochondrial ATP production, modulate ROS, and influence inflammatory and profibrotic signaling pathways [19, 20]. Differences in clinical response appear to reflect wavelength-specific penetration depth, with red light primarily affecting more superficial dermal layers and near-infrared light reaching deeper fibroblastic and vascular networks [19–21]. Improvements in pain and pruritus reported across multiple trials likely relate to PBM-mediated reductions in inflammatory cytokines and normalization of peripheral nerve signaling [6, 19, 22]. These mechanistic distinctions support a wavelength-tailored approach in which red LEDs are used for early and more superficial scars, and near-infrared LEDs for thicker, deeper, and more mature scars. Based on the clinical patterns observed across studies, wavelength-specific considerations for PBM in scar management are summarized in Table 3.

For early, actively remodeling scars, particularly burn scars and recent post-surgical incisions, red LED in the 633–670 nm range appears most appropriate, with moderate fluence levels favored to avoid the diminished response seen with excessively high doses. In contrast, deeper, thicker, or more mature scars, including hypertrophic scars older than one year and post-operative scars located on the trunk or deeper tissue planes, may benefit more from near-infrared wavelengths (808–833 nm), which achieve greater dermal penetration and modulate fibroblast activity beyond the superficial layers. Near-infrared LED can be integrated into postoperative care beginning one week after surgery or applied to longstanding scars, with improvements reported even in lesions older than 24 months old. Across all scar types, PBM should be viewed as an adjunct rather than a replacement for established therapies such as silicone sheeting, intralesional corticosteroid injections, pressure therapy, pulsed dye laser, or fractional ablative and non-ablative laser resurfacing. Selecting wavelength and fluence according to scar age, depth, and phenotype provides a rationale framework for PBM application alone or combined with other standard therapies pending standardized guidelines. Based on the patterns observed across clinical trials, wavelength-specific considerations for PBM in scar management are summarized in Table 3.

Despite promising clinical results, the integration of PBM into routine scar management remains limited. One challenge is the variability in available devices, wavelengths, and manufacturer-specific treatment parameters, which may create uncertainty among clinicians regarding optimal protocols. Standardization of PBM dosing parameters, treatment schedules, and device characteristics will likely be necessary before widespread adoption can occur. Until such consensus guidelines emerge, PBM may be best considered as an adjunctive modality used alongside established scar therapies within experienced clinical settings.

However, the available evidence remains limited by small sample sizes, heterogeneity in devices and treatment parameters, variable outcome measures, and short follow-up durations in several studies. The studies included in this review also evaluated heterogeneous patient populations and surgical contexts, which may limit generalizability across different scar types and anatomical locations. Variability in outcome assessment tools, including the VSS, POSAS, and subjective patient-reported measures, further complicates cross-study comparisons. Additionally, cumulative PBM exposure varied substantially across trials, ranging from approximately 52 to 384 J/cm^2^, reflecting considerable variability in dosing protocols. Outcomes in darker Fitzpatrick phototypes, keloid-prone individuals, and scars on high-tension anatomical areas with increased risk of hypertrophic or keloid scars formation also remain underexplored. Finally, the absence of standardized fluence, irradiance, and treatment frequency across studies highlights the need for rigorously designed, adequately powered randomized controlled trials.

Future research should prioritize adequately powered randomized trials that directly compare wavelengths, fluence ranges, and treatment timing across different scar etiologies. Incorporating objective imaging modalities such as three-dimensional surface analysis, would improve consistency across studies. Trials enrolling diverse phototypes and keloid-forming populations are particularly needed, as are comparative studies evaluating PBM alongside silicone therapy, intralesional corticosteroids, and fractional or vascular lasers. Ultimately, establishing consensus guidelines for LED and LLLT parameters will be essential for standardized clinical adoption.

## Conclusion

Red and near-infrared PBM represents a safe, non-invasive adjunct for scar management. Current evidence suggests red wavelengths may be most effective for early, superficial scars, whereas near-infrared wavelengths may offer greater benefit for deeper or more mature scars. Although treatment parameters remain heterogenous, wavelength selection based on scar age and depth provides a rational framework for clinical use. Further, parameter-controlled trials are needed to establish the standard for this promising therapy.

## Data Availability

No datasets were generated or analysed during the current study.
